# Transforming Brain Tumor Care: The Global Impact of Radiosurgery in Multidisciplinary Treatment Over Two Decades

**DOI:** 10.1002/cam4.70673

**Published:** 2025-03-14

**Authors:** Zerubbabel K. Asfaw, Tirone Young, Cole Brown, Mehek Dedhia, Lily Huo, Kunal K. Sindhu, Stanislav Lazarev, Robert Samstein, Sheryl Green, Isabelle M. Germano

**Affiliations:** ^1^ Department of Neurosurgery Icahn School of Medicine at Mount Sinai New York New York USA; ^2^ Department of Radiation Oncology Icahn School of Medicine at Mount Sinai New York New York USA

**Keywords:** Gamma Knife, global neurosurgery, linear accelerator, neuro‐oncology, neurosurgery, radiation oncology, stereotactic radiosurgery

## Abstract

**Background:**

Stereotactic radiosurgery, a minimally invasive treatment delivering high doses of radiation to a well‐defined target, has transformed interdisciplinary treatment paradigms since its inception. This study chronicles its adoption and evolution for brain cancer and tumors globally.

**Methods:**

A systematic literature review of SRS‐focused articles from 2000 to 2023 was conducted. Literature impact was evaluated using citation counts and relative citation ratio scores. Extracted data were dichotomized between US and international publications.

**Results:**

Out of 5424 articles eligible, 538 met inclusion criteria reporting on 120,756 patients treated with SRS for brain cancer and tumors since 2000. Over time, publication rates grew significantly (*p* = 0.0016), with 56% of principal investigators based in the United States. Clinical articles accounted for 87% of the publications, with the remainder focused on technological advances. Relative to international studies, US publications had larger median samples (74 vs. 58, *p* = 0.012), higher median citations (30 vs. 19, *p* < 0.0001) and higher relative citation ratio scores (1.67 vs. 1.2, *p* < 0.00001). Gamma Knife and LINAC had roughly equal representation in US and international publications. Neurosurgery specialists authored more Gamma Knife‐based articles, and radiation oncology specialists authored more LINAC‐based papers (*p* < 0.0001). The most treated tumors were metastases (58%), skull base tumors (35%), and gliomas (7%). Radiographic control was achieved in 82% of metastatic tumor cases, with a 12% median complication rate.

**Conclusions:**

SRS has been widely adopted both nationally and globally and continues to be a growing field. This study corroborates the clinical efficacy of SRS and reinforces its critical role in the multidisciplinary treatment of patients with brain tumors and cancer.

## 1 Introduction

Stereotactic radiosurgery (SRS) is a minimally invasive form of radiotherapy that uses three‐dimensional imaging and highly conformal radiation delivery to target small tissue regions precisely. Since its development in 1951, SRS has been utilized to treat a wide range of neurological disorders and pathologies [1]. The development of SRS was grounded in earlier scientific and technological advancements, such as the discovery of X‐rays and stereotaxis in the late 19th and early 20th centuries. More recent improvements in neuroimaging technology from the 1960s to the 1980s enabled the refinement of SRS techniques used in clinical practice today [2].

SRS utilizes ionizing radiation from two primary sources: gamma rays and x‐rays. Gamma rays are delivered via a frame‐based Gamma Knife (GK) platform, first introduced in 1968. X‐rays, meanwhile, are delivered through a linear accelerator (LINAC), whose application for SRS was developed in the 1980s [3, 4]. These technological advances have allowed LINAC‐based SRS platforms to become increasingly common both in the United States and worldwide [5, 6]. The versatility and efficacy of SRS, which was originally designed to treat brain tumors and perform functional ablations, have allowed for a broadening of its use in the treatment of trigeminal neuralgia, epilepsy, and metastatic brain cancers [7, 8, 9].

SRS, as its name connotes, combines the precision of surgery with the benefits of radiation therapy [10], traditionally combining expertise in both neurosurgery and radiation oncology to ensure that the procedure is safe, effective, and tailored to the patient's needs. The collaboration between neurosurgeons and radiation oncologists integrates insights from anatomy and radiation planning, optimizing treatment outcomes while minimizing risks [11, 12]. Additionally, the decision to treat a patient with SRS is typically finalized through multidisciplinary discussion at weekly tumor boards harboring neuro‐oncologists, medical oncologists, neuropathologists, and neuro‐radiologists, making this a true multidisciplinary treatment [11]. This teamwork ensures comprehensive care from diagnosis to treatment planning, delivery, and post‐procedure monitoring, enhancing patient safety and providing a holistic treatment experience.

In 2000, the International Stereotactic Radiosurgery Society was established, a testament to the growing recognition of the utility of SRS. Since then, the utilization of SRS has accelerated worldwide. However, many aspects of its use remain under‐documented. These include technological advancements, safety and effectiveness improvements, treatment practice trends, and disparities in accessibility and utilization. A clearer understanding of these factors could reveal opportunities for improvement and further research. This study aims to chronicle the adoption and evolution of SRS for brain cancer and tumors in the United States and globally since 2000, emphasizing technical progress and assessing its impact on both national and international levels.

## 2 Methods

### 2.1 Systematic Literature Review

Adhering to Preferred Reporting Items for Systematic Reviews (PRISMA) guidelines for a systematic review, a literature search was conducted on PubMed and Embase databases for articles published from January 1, 2000 to December 31, 2023 [13]. All articles relevant to SRS were found by following a published guide for searching MEDLINE using Medical Subject Headings (MeSH) terms and exclusion of non‐human studies [14]. The following keywords and Boolean operators were utilized in the search strategy of all databases: “stereotactic radiosurgery,” “radiosurgery,” “stereotactic radiation therapy,” “neurosurgical procedures,” “brain,” “cranial,” and “spine.” A detailed search strategy is provided in Data S1. Search results were imported into Covidence systematic review software (Veritas Health Innovation, Melbourne, Australia). The inclusion criteria included: (1) original articles on human subjects or technical reports, (2) clinical use of SRS, (3) cranial neurosurgery articles, (4) publications pertinent to radiation oncology and related fields, such as nuclear medicine and medical physics, (5) full‐text availability, and (6) English language manuscripts. The exclusion criteria included: (1) articles on whole‐body radiation therapy; (2) reviews, commentaries, or conference proceedings; and (3) case reports or articles with less than five patients. Four authors (Z.K.A., T.Y., C.B., and L.H.) independently screened eligible manuscripts based on abstract and title, and the corresponding author (I.M.G.) resolved disagreements. The first four authors independently extracted data from the included articles through a blinded process.

### 2.2 Data Extraction

The extracted data consisted of standard elements of a citation, type of SRS technology, study cohort size, study design, outcome metrics, funding sources, and senior author's specialty, affiliation, and country. The prevalence of low‐ and low‐middle‐income countries (LIC and LMIC) was assessed based on the World Bank classifications [15]. The senior author of each publication was considered the principal investigator (PI). Multicenter studies in the United States and international locations were categorized under the PI's country. Outcome metrics, including radiographic control, complication rates, progression‐free survival (PFS), and overall survival (OS), were pooled from all eligible studies that reported these data. However, no formal analysis of heterogeneity was conducted due to variability in study designs, sample sizes, and reporting standards, which may have influenced cross‐study comparisons. Outcome data were dichotomized for patients with metastatic brain tumors (patients with cancer) and skull base tumors, including meningiomas, vestibular schwannomas, and pituitary adenomas.

### 2.3 Literature Impact Analysis

The total number of citations for all studies was obtained from Scopus or Google Scholar [16, 17]. The relative citation ratio (RCR) of each eligible manuscript was queried from the National Institutes of Health (NIH) iCite website to evaluate the influence of each article relative to other NIH‐funded studies [18]. RStudio version 4.3.1 (Boston, MA) was used to query and compile the Altmetric score for all articles to determine the attention garnered by an article in non‐academic domains [19].

### 2.4 Statistical Analysis

All categorical data were reported as percentages, while continuous data were reported as medians with an interquartile range [IQR] due to the non‐normal distribution of data. The Mann–Whitney *U*‐test was conducted to analyze the differences between US and international publications. The Spearman test was utilized to assess the significance of publication trends across decades. The proportion of categorical data was evaluated using a chi‐squared test. Data were analyzed using Microsoft Excel (Seattle, WA) and RStudio version 4.3.1 (Boston, MA). Tableau 2022.4 (Seattle, WA) was used for geospatial analysis and geographic visualizations. A *P*‐value of < 0.05 was considered statistically significant.

### 2.5 Results

#### 2.5.1 SRS Patient Population and Geographic Distribution

**FIGURE 1:**
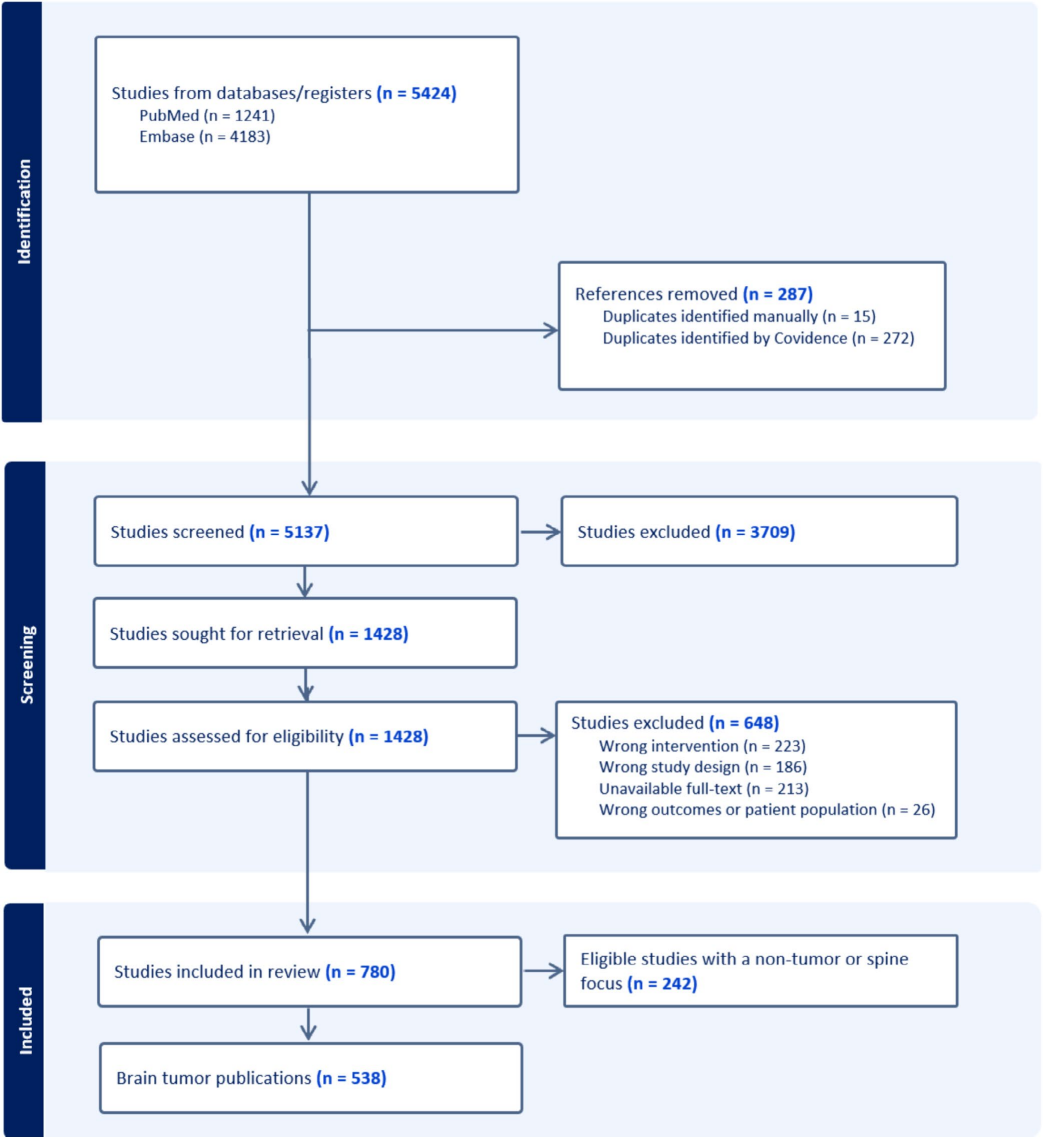
PRISMA summary and schematic representation of the systematic literature review of manuscripts pertinent to stereotactic radiosurgery for the treatment of brain tumors.

**FIGURE 2:**
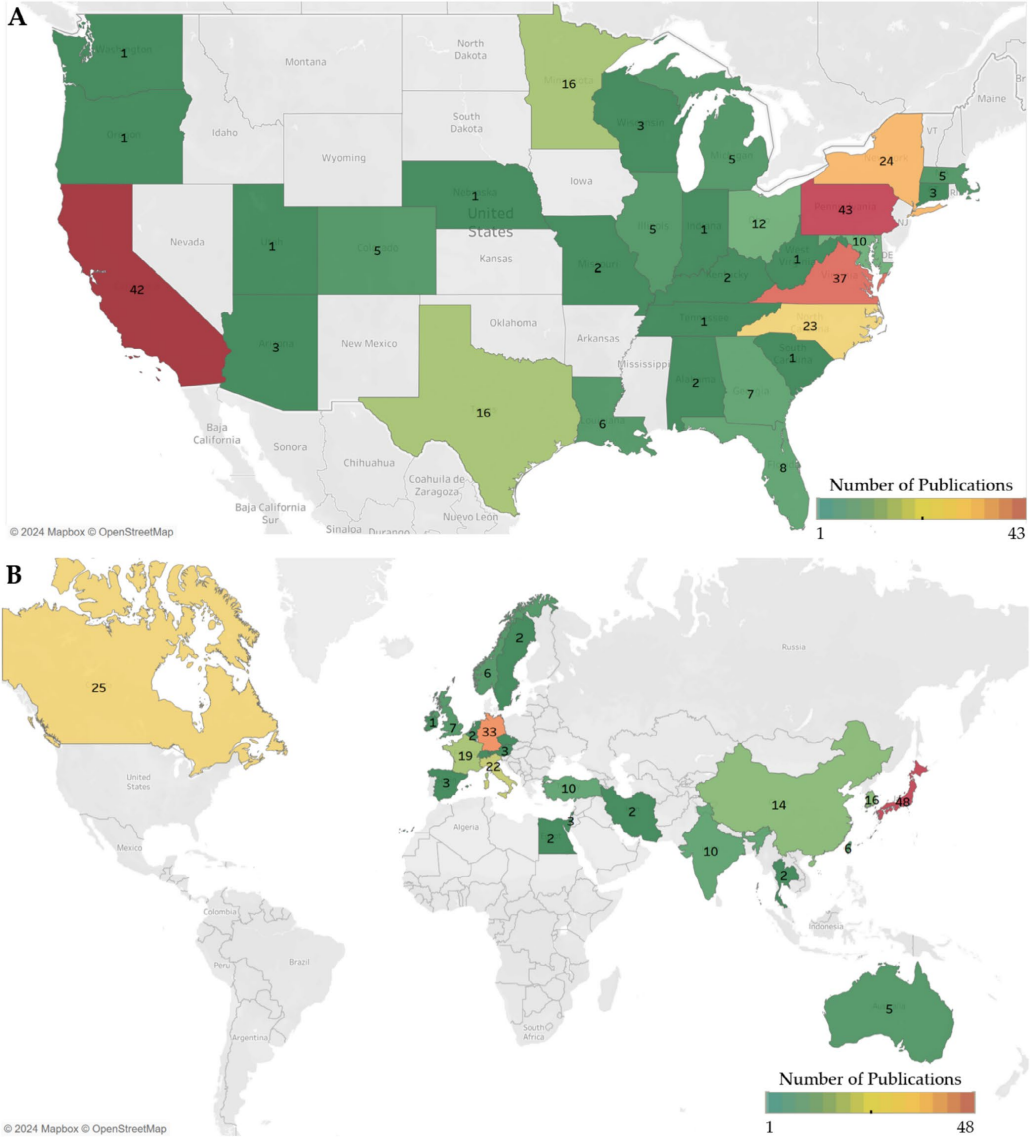
Geographic distribution of articles on stereotactic radiosurgery for the treatment of brain tumors in (A) the United States and (B) worldwide (excluding the US).

Figure 1 depicts the PRISMA summary from the initial 5424 articles eligible for screening to the 538 papers included in this literature review. These articles reported on 120,756 patients with brain tumors and brain cancer treated with SRS. Publications from the United States had a higher median sample size than international studies (72 vs. 58, *p* = 0.012). Approximately half of the SRS publications originated from non‐US countries (46%) (Figure 2, Table 1). In the United States, the top five states with the most SRS publications on brain cancer/tumors were Pennsylvania (43), California (42), Virginia (37), New York (24), and North Carolina (23) (Figure 2A). The leading countries for SRS publications outside the United States were Japan (48), Germany (33), Canada (25), Italy (22), and France (19). Notably, there were no articles from Latin America and the Caribbean, while Egypt was the only country represented from Africa (Figure 2B). Our cohort included three low‐ and middle‐income countries (LMIC) (Egypt, India, and Vietnam), which collectively produced 20 SRS articles during the study period; there were no publications from low‐income countries (LIC). Despite the relatively lower output, publication rates in LMIC increased over time, with nine articles published between 2018 and 2023 in prominent neurosurgery or radiation‐related journals.

**TABLE 1:** Summary of publications on the application of stereotactic radiosurgery for the treatment of brain tumors.

	All publications (*n* = 538)	US publications (*n* = 290)	International publications (*n* = 248)	*p* values
Citations, median (IQR)	25 (49)	30 (52)	19 (36.5)	**< 0.001**
RCR score, median (IQR)	1.44 (1.7)	1.67 (1.8)	1.21 (1.4)	**< 0.0001**
Altmetric score, median (IQR)	3 (5.4)	3 (6.0)	2.6 (4.4)	**0.007**
Total sample size (median, IQR)	120,756 (64, 118)	54,526 (72, 154)	66,230 (58, 93)	**0.012**
Study design				
Case series	157 (29.2)	87 (30.0)	70 (28.2)	0.15
Cohort studies	229 (42.6)	131 (45.2)	98 (39.5)	
Cross‐sectional	29 (5.4)	12 (4.1)	17 (6.9)	
Randomized controlled trial	24 (4.5)	11 (3.8)	13 (5.2)	
Non‐randomized trial	30 (5.6)	17 (5.9)	13 (5.2)	
Technical reports	66 (12.3)	29 (10.0)	37 (14.9)	
Other	3 (0.6)	3 (1.0)	0	
SRS technology				
Gamma Knife	322 (61.7)	181 (63.9)	141 (58.9)	0.37
LINAC	200 (38.3)	102 (36.0)	98 (41.1)	
Tumor type				
Metastatic tumors	266 (58.2)	139 (55.6)	127 (61.3)	0.48
Vestibular schwannoma	37 (8.1)	21 (8.4)	16 (7.7)	
Gliomas	34 (7.4)	24 (9.6)	10 (4.8)	
Meningioma	37 (8.1)	19 (7.6)	18 (8.7)	
Pituitary	30 (6.6)	19 (7.6)	11 (5.3)	
Other skull base tumors	53 (11.6)	28 (11.2)	25 (12.1)	

*Note:* *p* value < 0. 005 signifies a statistically significant difference calculated by Mann‐Whitney test as detailed in Methods.

**FIGURE 3:**
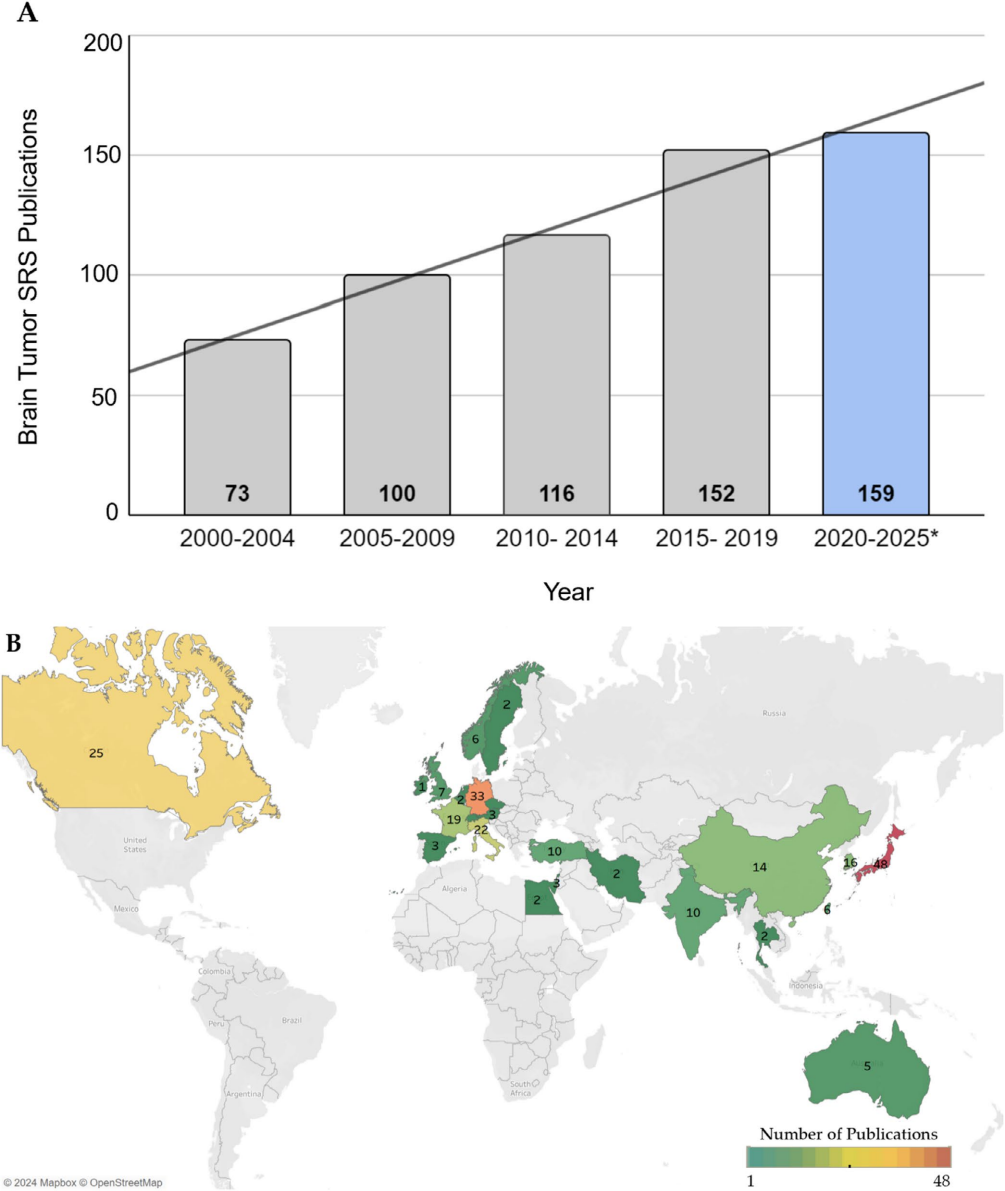
(A) Publication trend of articles on stereotactic radiosurgery (SRS) for the treatment of brain tumors from 2000 to 2025 (*r* = 0.61, *p* = 0.0016). (B) Publication trend of articles on stereotactic radiosurgery for the treatment of brain tumors based on tumor type of interest from 2000 to 2023.

Within the study period, there was a significant increase in the number of publications (*r* = 0.61, *p* = 0.0016), and a record number of publications per 5 years is projected to be achieved by 2025 (Figure 3A).

#### 2.5.2 SRS Impact on Academic and Practice Domains

The median number of citations for all publications was 25. US articles had a higher median number of citations (30 vs. 19, *p* < 0.001), median RCR score (1.67 vs. 1.21, *p* < 0.0001), and Altmetric score (3 vs. 2.6, *p* = 0.007) than international publications. The median RCR score of all publications was 1.44. Within non‐academic domains, the median Altmetric score of all studies was 3 (Table 1).

#### 2.5.3 SRS Study Design, Study Type, and Specialty Representation

Regarding study design, cohort studies followed by case series were the predominant designs for all articles. There were 54/583 (9.2%) clinical trials, of which 24 were randomized controlled trials. In terms of geographic distribution, 17/30 (56.7%) non‐randomized clinical trials and 11/24 (45.8%) randomized controlled trials were based in the United States, while international locations served as sites for 13 non‐randomized clinical trials and 13 randomized clinical trials. Among clinical trials, there were six multicenter and two single‐center phase III clinical trials. The single‐center trials were based in South Korea and the United States, while the multicenter trials were based in Canada, Germany, Japan, and the United States. Of the multicenter phase III clinical trials, one study recruited patients from more than one country—the United States and Canada.

Clinical studies constitute the majority of publications (87.7%), without a significant difference between those produced by the United States versus non‐US countries. Technical reports accounted for 12.3% of included studies in this review, with 55% originating from international sources. Faculty from Radiation Oncology and related fields were PIs for the majority of these publications, with neurosurgeons accounting for 29% and 28% of publications in the United States and abroad, respectively. While LINAC‐based articles constituted 66% of the international technical reports, GK‐based papers comprised 64% of US technical reports (*p* = 0.027). Common topics of focus for technical reports included dosimetric evaluations and treatment planning.

After stratifying studies based on the specialty of PI, dichotomized as radiation oncology and neurosurgery/neuro‐specialist, there was a significant difference in the distribution of the PI's country and SRS technology based on specialty (*p* = 0.031, Table 2). PIs specializing in neurosurgery and other neuro‐related fields were more predominant in both the US and non‐US countries than PIs specializing in radiation oncology.

**TABLE 2:** The distribution of the principal investigator's specialty based on country and stereotactic radiosurgery (SRS) technology.

		Principal investigator's specialty			
		Neurosurgery and neuro‐related	Radiation oncology	Other	*p*
Principal Investigator's Country	US	148 (51%)	140 (48%)	2 (1%)	Chi = 6.9554 , 0.031
International		137 (56%)	100 (41%)	8 (3%)	
SRS technology	Gamma Knife	219 (68%)	97 (30%)	6 (2%)	Chi = 65.17 > 0.0001
LINAC		63 (32%)	130 (66%)	4 (2%)	

#### 2.5.4 SRS Technology and Study Funding

Gamma Knife and LINAC technologies were represented without significant differences both within the United States and globally (Table 1). Neurosurgery and neuro‐related specialists authored more GK‐based articles, while radiation oncology specialists published more LINAC‐based articles (*p* < 0.0001, Table 2).

Study funding was reported in 10.2% of the publications. Governmental agencies supported 53% (29/55) of these studies. Foundations and Universities funded 27% (15/55), and private industry funded 20% (11/55). Elekta provided support to 46% of studies supported by Industry.

#### 2.5.5 SRS Patient Outcomes for Brain Cancer and Brain Tumors

The most frequent indication of SRS was metastatic disease regardless of geographic location (Table 1), without significant change over the study period (Figure 3B). Outcome data were extracted as specified in the methods and stratified based on tumor type. For patients with metastatic tumors (*N* = 84,900), the median follow‐up was 12 months. The median radiographic control rate was 82%, the median complication rate was 11%, the median PFS was 7 months, and the median OS was 11 months (Table 3).

**TABLE 3:** Outcomes based on tumor type treated by stereotactic radiosurgery.

Tumor type	Total number of patients	Outcome variable				
		Median radiographic control %, (IQR)	Median complications %, (IQR)	Median follow‐up months (Short term, Long term)	Median PFS months (IQR)	Median OS months (IQR)
Metastatic tumors	84,900	82	11	12 (2, 50)	7	11
Gliomas	1592	65	19	15 (2, 62)	7	13
Vestibular schwannoma	5169	93	7	44 (12, 105)	9	42
Meningioma	5155	91	13	47 (6, 124)	38	54
Pituitary	4804	90	22	51 (7, 120)	49	NA
Other skull base tumors	5705	88	9	47 (6, 139)	27	40

Among 35,856 patients with skull base tumors, vestibular schwannomas were the most commonly treated (*N* = 5169). With a median follow‐up of 44 months, these cases showed a 93% radiographic control rate and a 19% complication rate. The median PFS and OS for patients with vestibular schwannomas were 9 months and 42 months, respectively. The outcomes of skull base brain tumors are summarized in Table 3. For gliomas, radiographic control was obtained in 65% of cases, with a 19% complication rate.

## 3 Discussion

Our work offers a thorough examination of the evolution and impact of SRS in treating brain cancer and brain tumors over the past two decades. By analyzing data from over 120,000 patients and 538 peer‐reviewed publications worldwide, this study provides a comprehensive overview of the clinical efficacy, technological advancements, and global adoption of SRS. The findings highlight the significant role of SRS in the multidisciplinary treatment of brain tumors and its contributions to improving patient outcomes.

### 3.1 Clinical Efficacy of SRS in Brain Metastases

Our study underscores the clinical efficacy of SRS for brain metastases, achieving a median radiographic control rate of 82%, a median OS of 11 months, and a median PFS of 7 months. These outcomes affirm the role of SRS as a vital treatment modality for brain metastases. Although a direct comparison between SRS, open surgery, and systemic therapies is beyond the scope of this work, existing literature highlights SRS's advantages as a minimally invasive alternative with comparable or superior clinical outcomes in specific contexts.

SRS offers multiple benefits that enhance both clinical outcomes and patient quality of life. Unlike traditional surgical interventions, SRS is typically an outpatient procedure that avoids the need for hospital admission, thereby reducing associated risks, healthcare costs, and patient burden [10, 20]. The absence of hospitalization also minimizes indirect costs, such as lost wages due to extended recovery periods, making SRS a cost‐effective option from a socioeconomic standpoint [21]. Moreover, the ability to perform SRS without significantly interrupting ongoing systemic therapies, such as chemotherapy or targeted therapies, is particularly advantageous in maintaining treatment continuity, which is critical for optimal oncological control [22, 23].

SRS's precision in targeting metastatic lesions ensures that high‐dose radiation is confined largely to the tumor volume, thus sparing surrounding healthy brain tissue [20]. This precision not only enhances radiographic control but also reduces the risk of neurocognitive decline, a significant concern with alternative modalities of treatment like whole‐brain radiation therapy [24]. This attribute is especially relevant for patients with limited brain metastases, where preserving cognitive function and overall neurological integrity is a realistic outcome. Furthermore, SRS provides the flexibility to retreat recurrent or new metastases without cumulative toxicity, offering a durable control strategy that can be integrated into a patient's broader oncological management plan [25].

### 3.2 Long‐Term Outcomes and Complications in Benign Tumor Management With SRS

The higher complication rates observed in our study, particularly in patients with benign tumors like vestibular schwannomas, can be attributed to the extended survival of these patients, allowing late‐onset complications to manifest over time [26]. As patients with benign tumors typically have longer life expectancies, they are more likely to experience delayed adverse effects, with radiation‐induced cranial neuropathies often appearing several years post‐treatment [27]. Studies show that complications such as radiation necrosis, hemorrhage, tumefactive cysts, and hearing loss can emerge as late effects, sometimes several years following SRS [28, 29].

The evolution in SRS techniques has been crucial in mitigating these complications. Advancements, such as lowering the prescribed dose and the widespread adoption of cochlear dose constraints, have been shown to reduce the incidence of cranial nerve deficits. The 2018 Congress of Neurological Surgeons (CNS) guidelines highlight that, for single‐fraction SRS, a cochlear dose of less than 4 Gray (Gy) is associated with better hearing preservation [30]. Conversely, doses exceeding 4.2 Gy carry a higher risk of hearing loss, underlining the importance of precise dose management in maintaining the quality of life for these patients [31].

The trend toward adjusting radiation doses in SRS has significantly improved outcomes in patients with vestibular schwannomas, with the reduced rates of facial nerve palsy and trigeminal neuropathy [32, 33, 34, 35]. Current GK protocols typically use marginal doses of 12–13 Gy, optimizing tumor control while minimizing complications [36, 37]. Despite these advancements, late complications, such as cranial nerve dysfunction and hearing loss, can still occur due to delayed radiation effects, highlighting the importance of long‐term monitoring and individualized treatment strategies [38, 39, 40].

### 3.3 Areas of Potential SRS Expansion: Global Distribution and Study Design

The global rise in SRS utilization is driven by technological advancements, including improved imaging for precise targeting, advanced delivery systems like GK, CyberKnife, and LINAC, and less invasive frameless techniques supported by robotic systems [41, 42, 43, 44, 45, 46, 47]. Enhanced treatment planning software and real‐time tracking have further optimized dose delivery, reducing treatment times and improving patient outcomes [41, 48].

Our study found that SRS research is predominantly conducted in high‐income countries (HIC), with the United States leading in publication volume and academic impact. US studies demonstrated higher median sample sizes and citation metrics compared to international studies, indicating a greater academic influence. Researchers in LMIC face significant challenges, including economic barriers such as high article processing charges for open‐access publications, which limit their ability to publish in high‐impact journals [49, 50]. Furthermore, poorly defined regulatory and ethical guidelines, coupled with limited infrastructure, hinder their participation in global research initiatives [51]. Institutional barriers, such as visa restrictions, further restrict international collaboration, while unequal power dynamics in partnerships often marginalize LMIC researchers, diminishing their influence on research agendas [52].

The geographical distribution revealed significant contributions from countries such as Japan, Germany, Canada, Italy, and France, while publications from Latin America were absent. Only one country (Egypt) from Africa was represented in our sample. While the absence of publications does not directly imply a lack of SRS technology or expertise in LMIC and LIC, it underscores the significant challenges these regions face in accessing and maintaining the advanced infrastructure required for SRS. Our analysis revealed only 20 publications from LMIC, with 75% originating from India (15), followed by 20% from Egypt (4), and 5% from Vietnam (1). This limited representation suggests substantial barriers, including the high cost of equipment, scarcity of trained specialists, and inadequate institutional support [53, 54]. Additionally, it is plausible that SRS research is being conducted in these regions but is underrepresented in leading journals due to factors like language barriers, regional biases in academic publishing, and limited access to international collaborations that often facilitate publication in high‐impact journals [53, 54]. Previous studies have demonstrated that less than 5% of neurosurgery publications come from LMIC, despite these countries comprising nearly 80% of the global population [53]. Compounding these issues are systemic challenges such as limited research funding, underdeveloped data collection and analysis capabilities, and publication barriers in non‐English languages, which further reduce visibility in global literature [53]. This underrepresentation not only limits our understanding of the true landscape of SRS in these regions but also exacerbates global inequities in neurosurgical research, impeding the development of universally applicable treatment guidelines and strategies.

Efforts to address geographic disparities in SRS access, particularly in Latin America and Africa, are progressing through international collaborations such as the Global Radiosurgery Consortium [55]. Remote training programs have shown significant promise, with Sarria et al. demonstrating improved knowledge and confidence among radiation oncology practitioners in Latin America through longitudinal virtual education [55]. Technological upgrades, coupled with structured training initiatives, have also proven effective, as exemplified by the Sociedad de Lucha Contra el Cáncer in Ecuador [56]. Additionally, tele‐radiotherapy networks, as proposed by Datta et al., offer a scalable solution for resource sharing and capacity building [57].

Partnerships with high‐income countries and international organizations further bolster these efforts by providing essential funding, training, and technical support [58]. Addressing systemic socioeconomic and political barriers, as highlighted by Pannullo et al., remains critical to achieving equitable access to advanced radiotherapy technologies [59]. These multifaceted strategies collectively aim to close the gap in global SRS access and promote equity in neurosurgical care.

### 3.4 Leveraging Registries Over RCTs for Advancing SRS Research

While SRS is widely adopted for the multidisciplinary treatment of patients with brain tumors, the limited number of RCTs highlights an opportunity for more rigorous research. Large‐scale, multicenter studies are needed to validate current findings, explore long‐term outcomes, and provide a more nuanced understanding of the clinical and quality‐of‐life impacts of SRS across diverse settings [60, 61, 62]. However, RCTs in this domain are constrained by several factors, including the substantial financial burden—averaging $47,000 per patient—and the logistical complexities of patient recruitment, particularly for rare conditions or invasive interventions [12, 63]. Additionally, the inherent challenges of accruing sufficient patient numbers in trials with stringent inclusion criteria and long follow‐up periods often render RCTs infeasible for answering key clinical questions in SRS [12, 64, 65].

The SRS registry, developed by the American Association of Neurological Surgeons and NeuroPoint Alliance, provides a cost‐efficient alternative for gathering high‐quality clinical data [66]. It systematically captures real‐world outcomes across institutions, aiming to define national care patterns, offer benchmark data for quality improvement, generate actionable treatment insights, and support comparative effectiveness research [66, 67]. Additionally, the registry provides longitudinal data essential for understanding long‐term outcomes, making it a valuable tool for advancing evidence‐based SRS practice [66, 67]. While the SRS registry offers numerous advantages, it also has limitations. One notable drawback is the challenge of accurately capturing treatment‐related toxicities in a registry‐based format. Unlike RCTs, which typically involve more controlled and frequent follow‐up assessments to monitor adverse effects, registries may lack the detailed, consistent documentation required to thoroughly assess toxicity. This could potentially lead to underreporting or delayed recognition of complications, thus limiting the depth of toxicity‐related insights.

From a methodological standpoint, the SRS registry outperforms traditional RCTs by enabling the capture of large‐scale, heterogeneous datasets across diverse patient populations and clinical settings. This allows for a more comprehensive and representative analysis of SRS practices compared to the tightly controlled environments typical of RCTs [67, 68]. By leveraging data collected from real‐world clinical workflows, the registry supports big data analytics and retrospective studies that can identify trends, optimize treatment protocols, and detect disparities in care [68]. Additionally, the registry's ability to aggregate and harmonize data across different SRS platforms ensures consistency and facilitates cross‐institutional comparisons, thereby offering insights that are both generalizable and applicable to everyday clinical decision‐making. Ultimately, this approach not only reduces the costs associated with large‐scale prospective studies but also accelerates the generation of clinically relevant knowledge that can improve patient outcomes on a global scale.

Although SRS has been widely adopted for the multidisciplinary treatment of patients with brain tumors, the limited number of RCTs underscores an opportunity for the field for further large‐scale, multicenter studies to validate current findings, explore long‐term outcomes, and provide a more nuanced understanding of the clinical and quality‐of‐life impacts of SRS beyond HIC borders [60, 61, 62].

### 3.5 The Critical Role of Multidisciplinary Collaboration

Our results underscore the distinct, yet complementary, roles that neurosurgeons and radiation oncologists play in the advancement and application of SRS. Neurosurgeons provide critical anatomical expertise and surgical precision, essential for targeting complex intracranial structures. On the other hand, radiation oncologists provide crucial expertise in dosimetry, radiation physics, and broader applications, including fractionated treatments and extracranial procedures.

This dynamic interplay between neurosurgery and radiation oncology is not just operational but foundational, ensuring that both neuroanatomical complexities and radiobiological principles are balanced to achieve optimal treatment outcomes [11, 12]. Clinically, this collaborative model drives innovation and standardization in SRS practices. Regular interdisciplinary discussions among neurosurgeons, radiation oncologists, neuro‐oncologists, neuroradiologists, and neuropathologists are crucial for refining techniques, minimizing complications, and enhancing patient outcomes [11, 69, 70]. The evolving literature in SRS increasingly reflects this multidisciplinary integration, with studies at the intersection of neuro‐oncology, radiation physics, and surgical innovation.

### 3.6 Study Limitations

There are notable limitations in this article. First, this study was limited to papers published after 2000. We chose 2000 as the starting point because it aligns with the most recent pioneering advancements in SRS and coincides with the recognition of its utility on the global stage, marked by the establishment of the International Stereotactic Radiosurgery Society [71]. Second, any articles not written in English were excluded, which might have limited the representation of the distribution of radiosurgery literature on a global scale. Additionally, while PubMed and EMBASE cover a broad range of peer‐reviewed journals, this review may have excluded articles from LMIC and non‐English‐speaking regions, particularly those published in non‐indexed journals and regional databases. Furthermore, variability in study designs and reporting standards across different countries could have introduced heterogeneity in the data, impacting cross‐study comparisons.

## Author Contributions

**Zerubbabel K. Asfaw:** conceptualization (equal), data curation (equal), formal analysis (equal), investigation (equal), methodology (equal), project administration (equal), visualization (equal), writing – original draft (equal), writing – review and editing (equal). **Tirone Young:** conceptualization (equal), data curation (equal), formal analysis (equal), investigation (equal), methodology (equal), project administration (equal), visualization (equal), writing – original draft (equal), writing – review and editing (equal). **Cole Brown:** data curation (equal), investigation (equal), writing – original draft (equal). **Mehek Dedhia:** data curation (equal), formal analysis (equal), writing – original draft (equal), writing – review and editing (equal). **Lily Huo:** data curation (equal), investigation (equal), writing – original draft (equal). **Kunal K. Sindhu:** formal analysis (equal), methodology (equal), validation (equal), writing – review and editing (equal). **Stanislav Lazarev:** formal analysis (equal), methodology (equal), validation (equal), writing – review and editing (equal). **Robert Samstein:** formal analysis (equal), methodology (equal), validation (equal), writing – review and editing (equal). **Sheryl Green:** formal analysis (equal), methodology (equal), validation (equal), writing – review and editing (equal). **Isabelle M. Germano:** conceptualization (equal), data curation (equal), formal analysis (equal), investigation (equal), methodology (equal), project administration (equal), supervision (equal), validation (equal), visualization (equal), writing – original draft (equal), writing – review and editing (equal).

## Conflicts of Interest

Isabelle M. Germano has a consulting role with Brainlab and Integra. The other authors have no potential financial or non‐financial interests to disclose. The remaining authors declare no conflicts of interest pertinent to the work presented in this report.

## Supporting information


Data S1.

## Data Availability

The data that support the findings of this study are available from the corresponding author upon reasonable request.
