# Supplementary material for: Transforming Brain Tumor Care: The Global Impact of Radiosurgery in Multidisciplinary Treatment Over Two Decades
Source: Cancer Med. 2025 Mar 14;14(6):e70673. doi: 10.1002/cam4.70673 (PMC11909010; doi:10.1002/cam4.70673)
Supplement: Supplementary file 1 — Data S1. [file CAM4-14-e70673-s001.docx]

**Supplement 1:** Review Search Strategy

**PubMed**

- Keywords**:**
  - Radiosurg*
  - Neurosurg*
  - Brain
  - Crani*
- **Search**: (“Radiosurgery”[MESH] OR Radiosurg* OR "Stereotactic Radiation Therapy") AND (“Neurosurgical Procedures”[MESH] OR Neurosurg* OR “Brain” [MESH] OR “Spine” [MESH] Brain OR Spine* OR Spinal OR Crani*)

**EMBASE**

- Keywords
  - Radiosurg*
  - Stereotactic Radiation Therapy
  - Neurosurgery
  - Brain
  - Spine
  - Crani*
- **Search**

1: exp radiosurgery/

2: (Radiosurg* or "Stereotactic Radiation Therapy").mp.

3: 1 or 2

4: exp neurosurgery/

5: exp brain/ 6 exp spine/

7: (Neurosurg* or Brain or Spine* or Spinal or Crani*).mp.

8: 4 or 5 or 6 or 7

9: 3 and 8

10: limit 9 to (full text and human and english language and "remove preprint records")

11: limit 10 to yr="2000 - 2023"

Filter: AND NOT ((INDEX TERMS(animals OR animal)) AND NOT (INDEX TERMS(humans OR human)))
